# S-Nitrosoglutathione Is Not a Substrate of OATP1B1, but Stimulates Its Expression and Activity

**DOI:** 10.3390/biom15030428

**Published:** 2025-03-17

**Authors:** Yulia V. Abalenikhina, Aleksey V. Shchulkin, Olga N. Suchkova, Pelageya D. Ananyeva, Pavel Yu. Mylnikov, Elena N. Yakusheva, Igor A. Suchkov, Roman E. Kalinin

**Affiliations:** 1 Department of Biological Chemistry, Ryazan State Medical University, 390026 Ryazan, Russia; 2 Department of Pharmacology, Ryazan State Medical University, 390026 Ryazan, Russia; 3 Department of Cardiovascular, X-Ray Endovascular Surgery and Radiation Diagnostics, Ryazan State Medical University, 390026 Ryazan, Russia

**Keywords:** S-Nitrosoglutathione, OATP1B1, NO-cGMP signaling pathway, LXR*a*, Nrf2

## Abstract

S-nitrosoglutathione (GSNO) is the S-nitrosated derivative of glutathione (GSH). GSNO is an endogenous class of NO donors and a natural NO depot in biological systems. Organic anion transporting polypeptide 1B1 (OATP1B1) is an influx transporter that is expressed in the liver. OATP1B1 plays an important role in the transport of endogenous and exogenous substances. Various pathways for the regulation of OATP1B1 have been described. In the present study, the involvement of OATP1B1 in GSNO transport and the regulation of OATP1B1 by GSNO was examined. For HEK293-OATP1B1, it has been shown that GSNO is not a substrate of OATP1B1, but OATP1B1 can participate in the transport of GSH across the cell membrane. GSNO at concentrations of 1–100 μM and exposure for 3 h do not affect the expression and activity of OATP1B1, but exposure for 24 and 72 h stimulates the expression of the *SLCO1B1* gene, OATP1B1, and transporter activity. Up-regulation of OATP1B1 by GSNO is carried out through the NO-cGMP signaling pathway, Nrf2, and LXR*a*.

## 1. Introduction

S-nitrosoglutathione (GSNO) is the S-nitrosated derivative of glutathione (GSH). GSNO is an endogenous class of NO donors and a natural NO depot in biological systems [1]. NO is a biologically active molecule that regulates numerous physiological processes, for example, vascular tone, neurotransmission, immune system functioning, and it exhibits antioxidant and anti-inflammatory activity [2,3,4,5]. The NO-sGC-cGMP (nitric oxide II-soluble guanylate cyclase-cyclic guanosine monophosphate) signaling cascade is a classical mechanism for transmitting signals from NO into cells. It is carried out through a heme-containing protein—NO-sensitive soluble guanylate cyclase (sGC). GC catalyzes the conversion of guanosine triphosphate (GTP) into 3′,5′-cyclic guanosine monophosphate (cGMP) and pyrophosphate. Further initiation of signaling pathways in the cell proceeds through protein–protein interactions. The targets of cGMP are three classes of proteins. These are serine and/or threonine specific kinases: protein kinase G (PKG-1, PKG-2), cGMP-regulated phosphodiesterase (PDE), and ion channels [6].

NO can freely pass through the cell membranes. Due to its high reactivity, NO is rapidly oxidized in the intracellular environment and does not live long in a free state—its half-life is only 3–5 s [7]. This limits the effect of NO as a modulator of signaling pathways within the cell. However, there is a special class of NO donors that protect the NO group from oxidation and, at the same time, expand its temporal and spatial action [8]. Compared to other NO donors (organic nitrates, metal nitrosyl complexes, etc.) [9,10], GSNO is an endogenous molecule and a better candidate to the NO donor. In comparison to traditional ones, GSNO has no tolerance phenomenon and low toxicity [11,12]. At the cellular level, GSNO is in homeostatic balance with S-nitrosylated proteins. For example, GSNO reductase can change the amount of S-nitrosylated proteins in the cell and their associated signals through the catabolism of GSNO [13,14].

GSNO does not enter the cell directly. Initially, GSNO was supposed to decompose in the extracellular space releasing NO, which can diffuse across the cell membrane [15]. However, it was later found that the major mechanism of GSNO cellular uptake requires the transfer of the nitroso group from GSNO to another thiol-containing amino acid. After that, GSH and new S-nitrosothiols are transported into cells by special transport systems [1].

Membrane transporters play an important role in communication between the cell and the environment. Solute carrier (SLC) transporters are the largest membrane transport group in humans that includes over 400 members belonging to 66 families. Solute carrier (SLCs) transporters mediate the transport of a broad range of solutes across biological membranes [16]. Organic anion transporters (OATs), organic anion-transporting polypeptides (OATPs), organic cation transporters (OCTs), organic cation and carnitine transporters (OCTNs), and multidrug and toxin extrusion proteins (MATEs) are members of the SLC family that take part in the transport of xenobiotic drugs [17]. GSNO is considered to be a promising drug. To increase the effectiveness and safety of its use and to predict interactions with other drugs, it is necessary to test its affiliation with the substrates or inhibitors of clinically significant transporters [18]. The present study assessed the membership of the NO donor GSNO as substrates and modulators of organic anion transporting polypeptide 1B1 (OATP1B1). OATP1B1 is an anion uptake transporter that is mainly expressed on the basolateral membrane of liver cells [19]. OATP1B1 mediates the uptake of bile acids, thyroid hormones, steroid conjugates, and some drugs (statins, sartans, methotrexate) from blood to the liver [20].

The mechanisms of OATP1B1 regulation are being actively studied [21,22]. For example, some drugs, such as gemfibrozil, cyclosporine A, rifampicin, clarithromycin, and erythromycin, decrease the transporter activity due to their interaction with its molecule [23]. The expression of its gene (*SLCO1B1*) plays an important role in the regulation of OATP1B1 [22]. LXR*a* and FXR are nuclear receptors that are activated by oxysterols and bile acids, respectively. These nuclear receptors play essential roles not only in the regulation of cholesterol and bile acid metabolism, but also in sterol, fatty acid, and glucose metabolism [23]. LXR*a* and FXR are the main transcriptional regulators of OATP1B1 [24].

Nrf2 is a redox-sensitive transcription factor [25]. Normally, Nrf2 is associated with the repressor protein Keap1. Keap1 facilitates ubiquitination and proteasomal degradation of Nrf2 and prevents Nrf2 transport from the cytoplasm to the nucleus. The Keap1-Nrf2 complex dissociates after activation, and Nrf2 translocates to the nucleus and binds to the antioxidant responsive element (ARE) [26]. It was shown that NO is able to induce Nrf2–Keap1 signaling. NO triggered Nrf2 rapid nuclear accumulation, transcriptional activation and up-regulation of heme oxygenase 1, NAD(P)H dehydrogenase, and glutamate cysteine ligase [27]. Nrf2 activation is accompanied by increased expression of OATP1B1 [28].

The present study assessed the effect of the NO donor GSNO on OATP1B1 expression and activity, the role of the NO-cGMP signaling pathway, and the transcription factors Nrf2, FXR, and LXRa in this process, as well as the participation of OATP1B1 in the transmembrane transport of GSNO.

## 2. Materials and Methods

### 2.1. HepG2 Cell Line

HepG2 cells (Institute of Cytology, Russian Academy of Sciences, Russia) were cultured in Dulbecco’s modified Eagle’s medium (DMEM, Paneco, Moscow, Russia) with 15% fetal bovine serum (Biowest, Nuaille, France), 2 mM L-glutamine, 100 units/mL penicillin G, and 100 μg/mL streptomycin (all components manufactured by Paneco, Russia) at 37 °C in 5% CO_2_.

### 2.2. HEK293 and HEK293-OATP1B1 Cell Line

HEK293 cells (Institute of Cytology, Russian Academy of Sciences, Russia) were cultured in DMEM with 10% (*v*/*v*) fetal bovine serum, 2 mM L-glutamine, 100 units/mL penicillin G, and 100 μg/mL streptomycin at 37 °C in 5% CO_2_. HEK293 cells were stably transfected with the pEGFP-SLCO1B1 plasmid [29].

### 2.3. Assessment of GSNO’s Affiliation to OATP1B1 Substrates

HEK293 and HEK293-OATP1B1 were cultured on a 24-well plate. Cells were washed with uptake buffer (Hanks’ Balanced Salt Solution, 12.5 mM HEPES (pH 7,4) and 1% DMSO, Paneco, Russia). HEK293 and HEK293-OATP1B1 were incubated with the 1 and 10 μM GSNO (Sigma Aldrich, USA) solution at room temperature for 5, 15, and 30 min. The reaction was stopped by removing the uptake buffer and adding ice-cold uptake buffer to the well. Then, the cells were washed three times with 0.5 mL of ice-cold Dulbecco’s phosphate-buffered saline (Paneco, Russia). The cells were lysed by three “freeze-thaw” cycles. The concentration of GSNO in HEK293 and HEK293-OATP1B1 cells was compared. The uptake of 1 μM atorvastatin (Sigma Aldrich, USA) (the substrate of OATP1B1) was used as a positive control.

### 2.4. Assessment the Effect of GSNO on OATP1B1 and Its Mechanisms

HepG2 cells were cultured in 6-well plates. GSNO was used at concentrations of 1, 10, 50, and 100 μM and incubated for 3, 24 and 72 h.

To test the role of the NO-sGC signaling pathway, nuclear factor erythroid 2-related factor 2 (Nrf2), farnesoid X receptor (FXR), and liver X receptor isoform *a* (LXR*a*) in the regulation of OATP1B1 by GSNO, we blocked them by specific inhibitors: the inhibitor of sGC—1H-[1,2,4]Oxadiazolo[4,3-a]quinoxalin-1-one - ODQ (Sigma-Aldrich, USA) at the concentration of 10 μM [30], the inhibitor of Nrf2—N-(1,3-benzodioxol-5-ylmethyl)-5-(4-fluorophenyl)-thieno [2,3-d]pyrimidin-4-amine—AEM1 (Sigma-Aldrich, USA) at the concentration of 5 μM [31], the inhibitor of FXR—tauro-β-cholic acid (β-TA) at the concentration of 200 μM (Sigma Aldrich, USA) [32], the inhibitor of LXR*a*—3-(3,4-Dimethoxyphenyl)-N-[4-(trifluoromethyl)phenyl]-2-propenamide, and N-(4-Trifluoromethylphenyl) 3,4-dimethoxycinnamide—TFCA (Sigma Aldrich, USA) at the concentration of 30 μM [33].

To study the effect of GSNO on OATP1B1 activity, HepG2 cells were cultured in 24-well plates. GSNO was added to the cells at concentrations of 1, 10, 50, and 100 μM and incubated for 3, 24, and 72 h. The control wells were supplemented with culture medium with an equivalent amount of millipore water (GSNO solvent). At the end of the incubation, the cells were washed with pre-warmed (37 °C) uptake buffer and incubated with the 1 μM atorvastatin solution at room temperature for 5, 15, and 30 min. The reaction was stopped by removing the uptake buffer and adding ice-cold uptake buffer. The cells were lysed by three “freeze-thaw” cycles.

Rifampicin, an OATP1B1 inhibitor (Sigma Aldrich, USA), at similar concentrations and with a 15 min pre-incubation, was used as a positive inhibition control.

### 2.5. GSNO and GSH LC-MS/MS Analysis

GSNO and GSH in the cell lysates were analyzed by LC-MS/MS using Ultimate 3000-TSQ Fortis with an electrospray ionization (ESI+) source (Thermo Fisher Scientific, USA). Proteins were precipitated by methanol (Chimmed, Moscow, Russia), 1:1, *v*/*v*. The separation was performed on the C18 column UCT Selectra (C18 4.6 mm × 100 mm, 3 um, 100 A) with pre-column Selectra C18 Guard Cartridges SLC-18GDC46-5UM, using formic acid (Panreac, Barcelona, Spain) solution (0.1%) as mobile phase A and methanol as mobile phase B. The mobile phase gradient was: 0–0.01 min—60% A and 40% B, 0.01–2.5 min—40% A and 60% B, 2.5–6.0 min—1% A and 99% B, 6.0–10.0 min—60% A and 40% B. The flow rate was 400 µL/min, the column temperature—35 °C. The injection volume was 10 μL. For the quantitative analysis, the optimized selective reaction monitoring (SRM) mode with the following parameters: for GSNO m/z 337.1 → 307.1 (was used for quantitative analysis), 337.1 → 202.1, for GSH m/z 308.1 → 179.1 (was used for quantitative analysis), 308.1 → 162.1 was performed.

### 2.6. Determination of the Nitric Oxide Level in HepG2 Cells

Determination of the nitric oxide level in HepG2 cells was performed using DAF-FM Diacetate (Thermo Fisher Scientific, USA) fluorescent probes [34]. Cells were visualized using the 74 Olympus CKX-53 microscope (Olympus, Japan). Then, the cells were removed from the wells and lysed using 0.2% Triton X-100 (Sigma-Aldrich, Germany). Quantitative assessment of the nitric oxide level in the cell lysates was determined by the degree of fluorescence (λext = 495 nm, λem = 515 nm). The analysis was carried out on a Shimadzu RF-6000 spectrophotometer (Shimadzu, Kyoto, Japan) and recalculated to the number of cells (Countess 3 Automated Cell Counter, Thermo Fisher Scientific, USA). The obtained values were expressed in fluorescence unit/10^6^ cells. Nuclei were stained with DAPI—the blue-fluorescent DNA stain.

### 2.7. Determination of Bityrosine Level in HepG2 Cells

To evaluate the degree of protein nitrosylation under the action of GSNO in HepG2 cells, the level of bityrosine cross-links was performed. In the cell lysate, fluorescence was recorded at the excitation wavelength of λext = 325 nm and the emission wavelength of λem = 415 nm [35].

### 2.8. Real-Time PCR

Expression of the *SLCO1B1* gene was verified by real-time PCR. Total RNA was extracted using the RNeasy Mini Kit (QIAGEN, Germany). Reverse transcription PCR (RT-PCR) was carried out with the RT-PCR SYBR Blue reagent kit (Biolabmiks, Novosibirsk, Russia). Reverse transcription conditions: temperature—45 °C, incubation time—10 min, number of cycles—1. The following primers were used: 5′-GGTGAATGCCCAAGAGATGATG-3′ (forward) and 5′-TGGAAACCCAGTGCA AGTGATT-3′ (reverse) (Evrogen, Moscow, Russia). Gene encoding glyceraldehyde dehydrogenase (GAPDH) was used as a reference gene with the following primers: 5′-GTCCCTCTGACTTCAACAGCG-3′ (forward) and 5′-ACCACCCTGTTGCTGTAGCCAA-3′ (reverse) (Evrogen, Russia). Analysis was performed under the following cycle conditions: denaturation—heating of the reaction mixture to 95 °C for 10 s, cooling at 53 °C for 10 s, and elongation—at 72 °C for 30 s. Number of cycles—40. Analysis was carried out with an Applied Biosystems Quant Studio 5 amplifier using hybridization fluorescent probes with real-time detection and the QuantStudio Design and Analysis program (Life Technologies Holdings Pte. Ltd., Singapore).

### 2.9. Western Blotting

To detect OATP1B1 expression, cells were lysed with NP40 Cell Lysis Buffer Thermo (Thermo Fisher Scientific, USA) with protease inhibitors (Sigma Aldrich, USA). To detect the Nrf2 level in the nuclear fraction of the cells, they were lysed with the ReadyPrep Protein extraction kit (Cytoplasmic/Nuclear) (Bio-Rad, Hercules, CA, USA) with protease inhibitors (Sigma-Aldrich, USA).

Protein concentration was quantified with the Pierce Coomassie Plus (Bradford) Assay Kit (ThermoFisher, USA). 20 μg protein/sample were separated by 10% SDS–PAGE and transferred onto nitrocellulose membranes using Transblot (Bio-Rad, USA). The membranes were blocked with TBS with 1% Casein Blocker (Bio-Rad, USA) for 1 h and then incubated overnight at 4 °C with primary antibodies (OATP2 Polyclonal Antibody, PA5-113548, Invitrogen, USA; AF0639 Nrf2 Polyclonal Antibody, Affinity; PAB863Ge01 Polyclonal Antibody to Nitrotyrosine (NT), Cloud-Clone, Wuhan, China). The membranes were washed in TBS (Bio-Rad, USA) and then incubated with secondary antibodies (Goat anti-Rabbit IgG (H+L) Cross-Adsorbed Secondary Antibody, HRP, Invitrogen, Carlsbad, USA) for 1 h at room temperature.

The OATP1B1 and nitrotyrosine expressions were measured relative to the level of GAPDH (primary antibodies—GAPDH Loading Control Monoclonal Antibody (GA1R), DyLight 68, Invitrogen, USA; secondary antibodies—Rabbit-anti-Mouse IgG (H+L) Secondary Antibody, HRP, Invitrogen, USA).

The Nrf2 level in the nuclear fraction was measured relative to the level of Laminin B1—the member of the nuclear lamin protein family (primary antibodies—Laminin B1, Affinity AF5161, China; secondary antibodies—Goat anti-Rabbit IgG (H+L) Cross-Adsorbed Secondary Antibody, HRP, Invitrogen, USA).

Detection was performed using ChemiDocXRS+ (Bio-Rad, USA). The intensity of the obtained bands was analyzed densitometrically using ImageLab 6.0.0 software (Bio-Rad, USA).

### 2.10. Atorvastatin LC-MS/MS Analysis

The concentration of atorvastatin in cell lysates was analyzed using a previously published method [36] with the Ultimate 3000-TSQ Fortis system and an electrospray ionization (ESI+) source (Thermo Fisher Scientific, USA).

### 2.11. Data Analysis

Statistical analysis was performed using GraphPad Prism 8.1.2. Data are presented as the mean (M) and standard deviation (SD). Differences were determined using ANOVA followed by Tukey’s or Dunnett’s multiple comparison test and unpaired *t*-tests. The value of *p *< 0.05 was considered statistically significant.

## 3. Results

### 3.1. GSNO Transport in HEK293-OATP1B1 and HEK293 Cells

**Figure 1:**
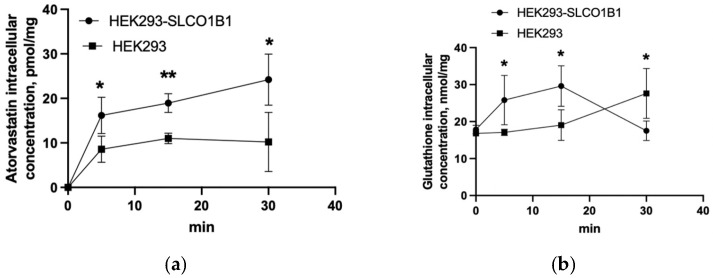
Atorvastatin (**a**) and GSH (**b**) uptake in HEK293-OATP1B1 and HEK293 cells. Atorvastatin was added to the cells at a concentration of 1 μM, GSNO (when detected GSH)—at a concentration of 10 μM. *—*p* < 0.05; **—*p* < 0.01—differences between uptake in HEK293-OATP1B1 and HEK293, Student’s *t*-test.

To estimate the participation of OATP1B1 in GSNO transport, GSNO uptake in HEK293-OATP1B1 and HEK293 cells was evaluated. GSNO was not detected in HEK293-OATP1B1 and HEK293 cell lysates when added at concentrations of 1 μM or 10 μM. At the same time, atorvastatin concentration in HEK293-OATP1B1 cell lysates was higher than the concentration in HEK293 at 5, 15, and 30 min (atorvastatin was added at the concentration of 1 μM) (Figure 1a). The data indicate that GSNO is not a substrate of OATP1B1. Interestingly, after the use of GSNO at a concentration of 10 μM, the concentration of GSH in HEK293-OATP1B1 cell lysates after 5 and 15 min of incubation was higher than in HEK293 cell lysates. After 30 min, on the contrary, the concentration of GSH was higher in HEK293 than in HEK293-OATP1B1 cell lysates (Figure 1b). These results indicate that OATP1B1 can participate in the transport of GSH across the cell membrane.

### 3.2. Effect of GSNO on the NO Levels in HepG2 Cells

**Figure 2:**
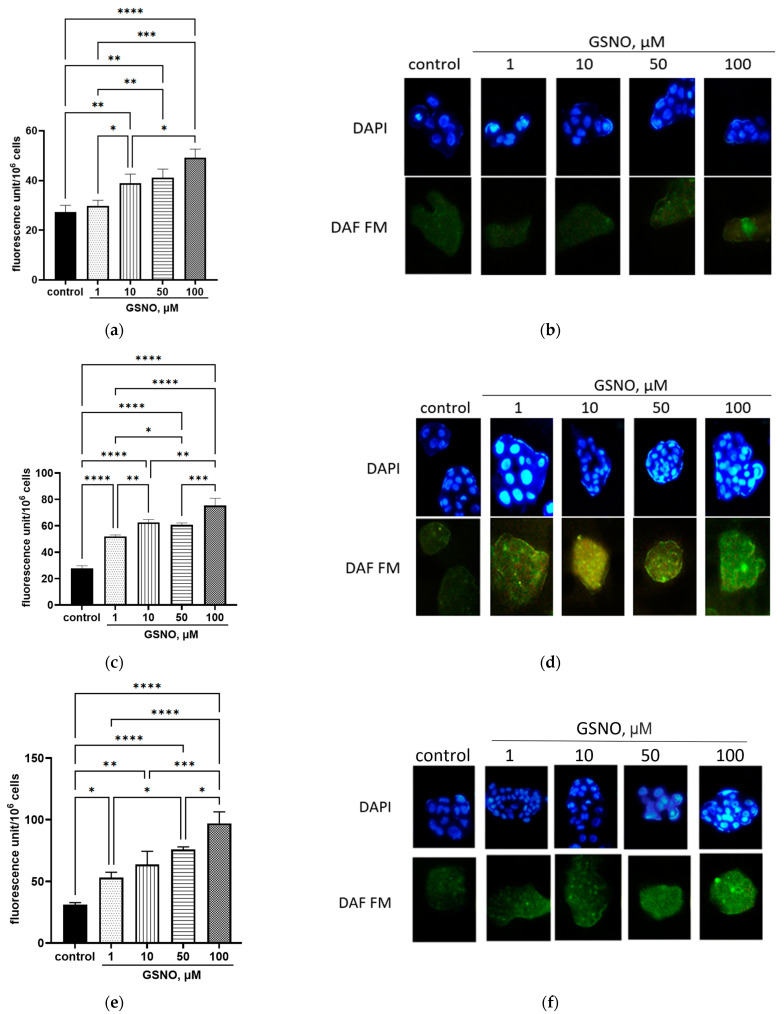
Fluorescence of the HepG2 cell lysates (**b**,**d**,**f**) and changes in the NO levels in the cells (**a**,**c**,**e**) after exposure to GSNO at concentrations of 1, 10, 50, and 100 μM for 3 h (**a**,**b**), 24 h (**c**,**d**), and 72 h (**e**,**f**) with DAF-FM staining. * *p* < 0.05; ** *p* < 0.01; *** *p* < 0.001; **** *p* < 0.0001—differences with the control, ANOVA, post-hoc Tukey’s test. (M ± SD, n = 3). Note: magnification ×400, nuclei staining with DAPI.

The NO level in the cells was determined using DAF-FM. 10–100 μM of GSNO increased the NO level with an exposure duration of 3 h. With exposure durations of 24 and 72 h, the NO level increased at the GSNO concentrations of 1, 10, 50, and 100 μM. At all incubation periods, the changes were dose-dependent—with an increase in the GSNO concentration, the fluorescence intensity (NO concentration) increased (Figure 2). The results indicate that GSNO is a donor of NO.

### 3.3. Effect of GSNO on the Expression of SLCO1B1 Gene

**Figure 3:**
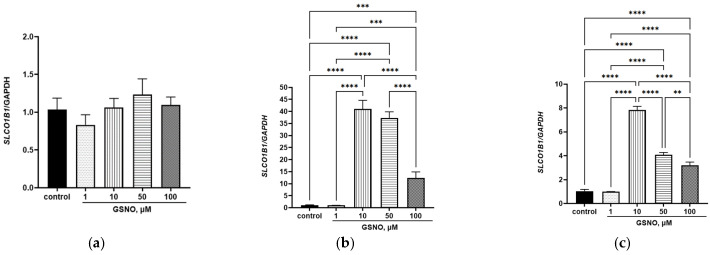
Effect of GSNO on the expression of *SCLO1B1* gene. Duration of exposure 3 (**a**), 24 (**b**), and 72 (**c**) h. ** *p* < 0.01; *** *p* < 0.001; **** *p* < 0.0001—differences with the control, ANOVA, post-hoc Tukey’s test. (M ± SD, n = 3).

GSNO at all concentrations did not affect the expression of the *SLCO1B1* gene at the exposure duration of 3 h. GSNO increased the expression of the *SLCO1B1* gene at the exposure durations of 24 h and 72 h and at concentrations of 10–100 μM (Figure 3). These changes were dose-dependent.

### 3.4. Effect of GSNO on the Expression of OATP1B1

**Figure 4:**
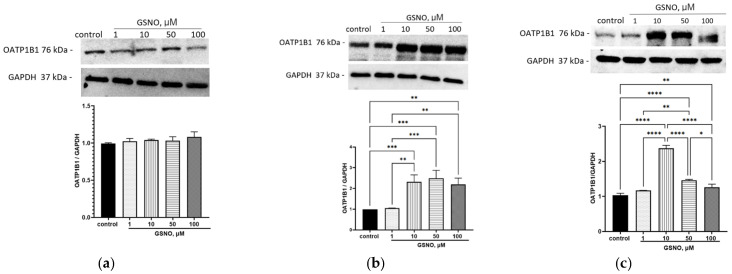
Effect of GSNO on the OATP1B1 expression. Duration of exposure 3 (**a**), 24 (**b**), and 72 (**c**) h. Results of western blotting and densitometric analysis of western blotting. * *p* < 0.05; ** *p* < 0.01; *** *p* < 0.001; **** *p* < 0.0001—differences with the control, ANOVA, post-hoc Tukey’s test. (M ± SD, n = 3). Original images can be found in Supplementary Materials.

GSNO at all tested concentrations (1–100 μM) and 3 h incubation did not affect the expression of OATP1B1. GSNO at concentrations of 10–100 μM and with exposure for 24 and 72 h increased the expression of OATP1B1, while at the concentration of 1 μM, it had no effect. The changes were dose-dependent (Figure 4). The obtained results indicate that GSNO up-regulates the expression of the *SLCO1B1* gene and OATP1B1.

### 3.5. The Effect of GSNO on the Intracellular Transport of the OATP1B1 Substrate Atorvastatin in HepG2 Cells

OATP1B1 activity in HepG2 cells was assessed by the uptake of the transporter substrate atorvastatin. GSNO did not affect the uptake of atorvastatin at the 3 h exposure time. At 24 h and 72 h exposures, GSNO at concentrations of 10 and 50 μM increased the uptake of atorvastatin into HepG2 cells. GSNO at concentrations of 100 μM did not affect the uptake of atorvastatin, despite the increase in OATP1B1 expression. Rifampicin, a classic OATP1B1 inhibitor, reduced the uptake of atorvastatin in HepG2 cells (Table 1).

### 3.6. The Role of NO-sGC Signaling Pathway in the Up-Regulation of OATP1B1 by GSNO

**Figure 5:**
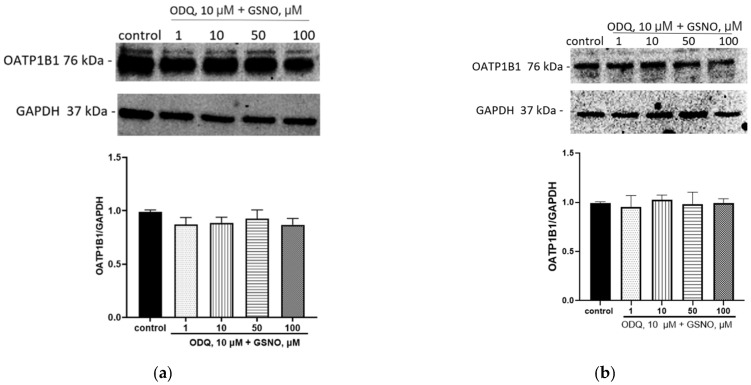
The role of NO-sGC signaling pathway in the induction of OATP1B1 by GSNO at concentrations of 10–100 µM and exposure for 24 h (**a**) and 72 h (**b**). Results of western blotting and densitometric analysis of western blotting. ANOVA, post-hoc Tukey’s test. (M ± SD, n = 3). Original images can be found in Supplementary Materials.

The inhibition of the NO-sGC signaling pathway by ODQ prevented the induction of OATP1B1 under the action of GSNO at concentrations of 10–100 µM and exposure for 24 and 72 h; its expression did not differ from the control (Figure 5). Thus, the effect of GSNO on OATP1B1 is mediated by the NO-sGC signaling pathway.

### 3.7. The Role of Nfr2 in the Up-Regulation of OATP1B1 by GSNO

**Figure 6:**
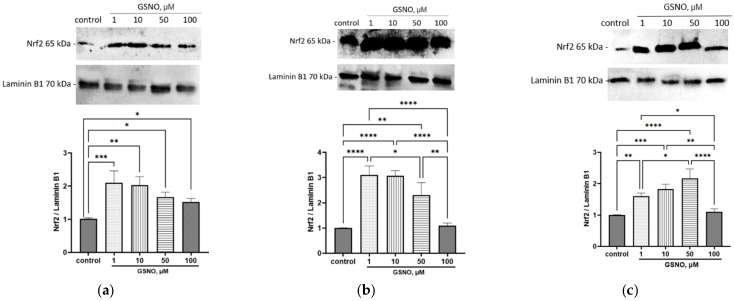
Effect of GSNO on the Nrf2 activation. Duration of exposure of 3 (**a**), 24 (**b**), and 72 (**c**) h. Results of western blotting and densitometric analysis of western blotting. * *p* < 0.05; ** *p* < 0.01; *** *p* < 0.001; **** *p* < 0.0001—differences with the control, ANOVA, post-hoc Tukey’s test. (M ± SD, n = 3). Original images can be found in Supplementary Materials.

GSNO at concentrations of 1–100 μM and exposure for 3 h caused an increase in the Nrf2 expression in the nuclear fraction of HepG2 cells. With exposure times of 24 and 72 h, GSNO at concentrations of 1–50 μM also caused an increase in the nuclear fraction of Nrf2; at a concentration of 100 μM, it had no effect (Figure 6). The obtained data indicate that GSNO can activate Nrf2.

**Figure 7:**
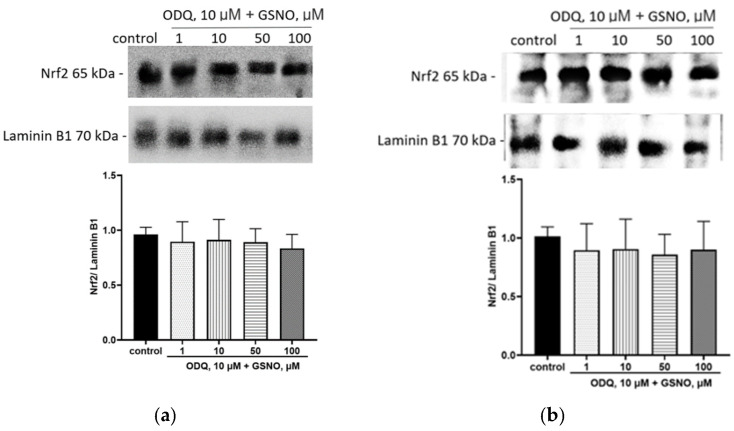
The role of NO-sGC signaling pathway in the up-regulation of Nrf2 by GSNO at concentration of 1–100 µM and exposure for 24 h (**a**) and 72 h (**b**). Densitometric analysis of western blotting and results of western blotting. ANOVA, post-hoc Tukey’s test. (M ± SD, n = 3). Original images can be found in Supplementary Materials.

Moreover, blocking of the NO-sGC signaling pathway prevented the Nrf2 activation by GSNO at concentrations of 1–100 µM and exposure for 24 and 72 h; its expression in nuclear fraction did not differ from the control (Figure 7). Thus, GSNO activates Nrf2 through the NO-sGC signaling pathway.

**Figure 8:**
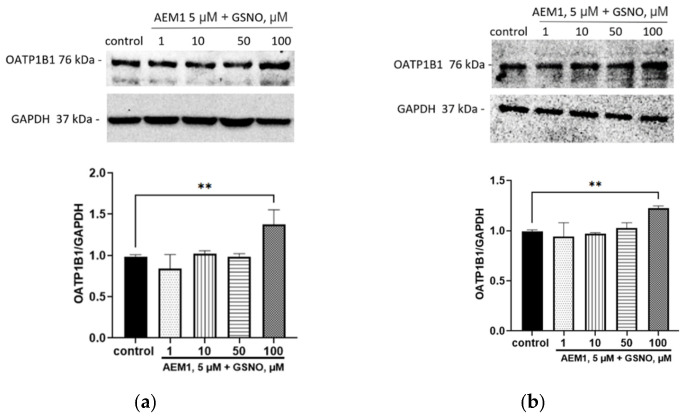
The role of Nrf2 in the up-regulation of OATP1B1 by GSNO at concentrations of 10–100 µM and exposure for 24 h (**a**) and 72 h (**b**). Densitometric analysis of western blotting and results of western blotting. ** *p* < 0.01—differences with the control, ANOVA, post-hoc Tukey’s test. (M ± SD, n = 3). Original images can be found in Supplementary Materials.

To determine the role of Nfr2 in the up-regulation of OATP1B1 by GSNO, we blocked it with AEM1. The inhibition of Nfr2 did not prevent the induction of OATP1B1 under the action of GSNO at a concentration of 100 µM and exposure for 24 and 72 h. The expression of the transporter protein exceeded the control. At the same time, the inhibition of Nfr2 prevented the up-regulation of OATP1B1 with the action of GSNO at concentrations of 10 and 50 µM and exposure for 24 h and 72 h (Figure 8).

### 3.8. The Role of FXR and LXRa in the Up-Regulation of OATP1B1 by GSNO

**Figure 9:**
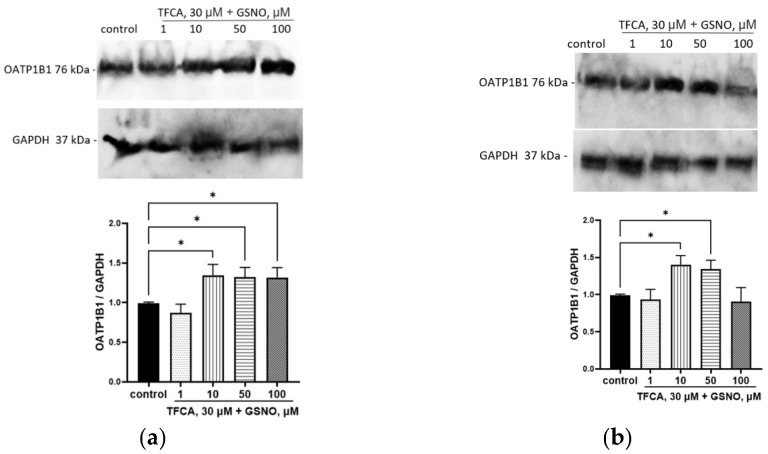
The role of LXR*a* in the up-regulation of OATP1B1 by GSNO at concentrations of 10–100 µM and exposure for 24 h (**a**) and 72 h (**b**). Densitometric analysis of western blotting and results of western blotting. * *p* < 0.05—differences with the control, ANOVA, post-hoc Tukey’s test. (M ± SD, n = 3). Original images can be found in Supplementary Materials.

**Figure 10:**
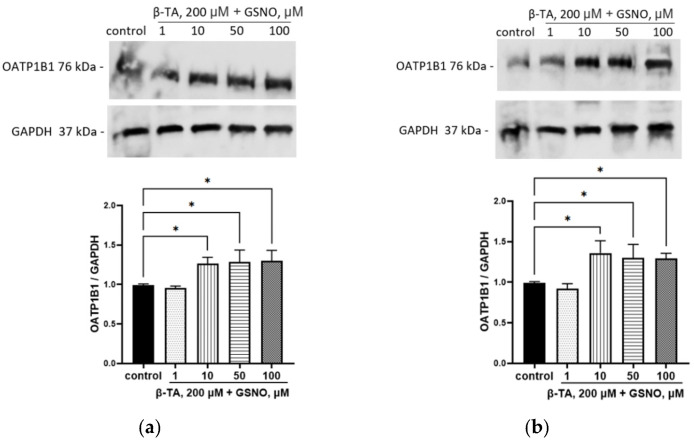
The role of FXR in the up-regulation of OATP1B1 by GSNO at concentrations of 10–100 µM and exposure for 24 h (**a**) and 72 h (**b**). Densitometric analysis of western blotting and results of western blotting. * *p* < 0.05—differences with the control, ANOVA, post-hoc Tukey’s test. (M ± SD, n = 3). Original images can be found in Supplementary Materials.

The inhibition of LXRa by TFCA did not prevent the induction of OATP1B1 by GSNO at concentrations of 10–100 µM with an exposure time of 24 h nor at the concentrations of 10–50 µM with an exposure time of 72 h. The inhibition of LXRa prevented the induction of OATP1B1 by 100 µM GSNO with an exposure time of 72 h (Figure 9). Inhibition of FXR had no effect on the up-regulation of OATP1B1 by GSNO; the expression of the transporter in all experiments exceeded control values (Figure 10).

### 3.9. Development of Nitrosative Stress Under the Action of GSNO

**Figure 11:**
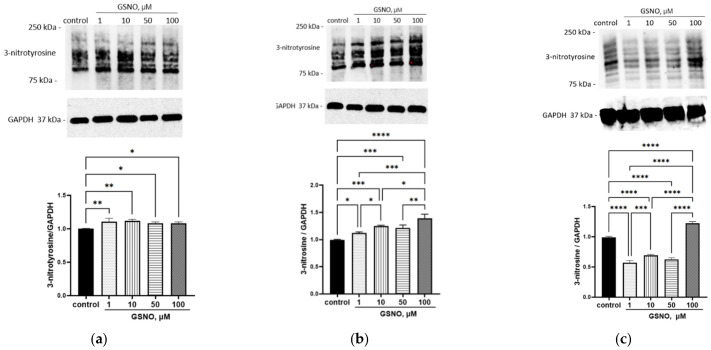
Effect of GSNO on the 3-nitrotyrosine level. Duration of exposure: 3 (**a**), 24 (**b**), and 72 (**c**) h. Results of western blotting and densitometric analysis of western blotting. * *p* < 0.05; ** *p* < 0.01; *** *p* < 0.001; **** *p* < 0.0001—differences with the control, ANOVA, post-hoc Tukey’s test (M ± SD, n = 3). Original images can be found in Supplementary Materials.

**Figure 12:**
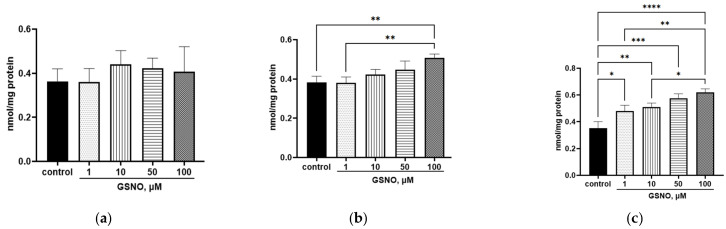
Effect of GSNO on the bityrosine level. Duration of exposure: 3 (**a**), 24 (**b**), and 72 (**c**) h. Results of western blotting and densitometric analysis of western blotting. * *p* < 0.05; ** *p* < 0.01; *** *p* < 0.001; **** *p* < 0.0001—differences with the control, ANOVA, post-hoc Tukey’s test. (M ± SD, n = 3).

The increase in NO level inside the cells triggered the nitrosylation process. When exposed to GSNO at concentrations of 1–100 μM and incubated for 3 and 24 h, the level of 3-nitrotyrosine increased (Figure 11). When exposed for 72 h to GSNO concentrations of 1–100 μM, the bityrosine level increased (Figure 12).

## 4. Discussion

GSNO is the main endogenous NO depot in cells and is considered a promising drug with NO-donating activity [1]. Therefore, the study of its biochemical and pharmacological effects is actively continuing. There are also some questions about the mechanism of its transport into cells. In this study, we assessed the belonging of GSNO to the substrates and modulators of OATP1B1. OATP1B1 is an influx liver transporter that ensures the penetration of substrates into hepatocytes. The substrates of this transporter are a wide range of endogenous substances, such as estrone-3-sulfate, bilirubin, etc. Therefore, we assumed that GSNO can also be transported into hepatocytes via OATP1B1 [21].

On the other hand, OATP1B1 is a transporter that plays an important role in the transport of drugs. Its substrates are such widely used drugs, such as statins, sartans, and angiotensin-converting enzyme inhibitors [20]. Therefore, if we consider GSNO as a therapeutic agent, it is necessary to know how it can affect OATP1B1 in order to predict the development of interactions with other drugs at the level of this transporter. The mechanisms of OATP1B1 regulation are being studied [22], and the role of NO and GSNO as its endogenous depot in this process has not been tested by this moment yet.

It has been shown that GSNO transport/penetration does not differ in HEK293 and HEK293-OATP1B1 cells (GSNO was not detected in either of them, despite the fact that the classical OATP1B1 substrate had a higher permeability in HEK293-OATP1B1). The obtained data indicate that GSNO is not a substrate for OATP1B1. It is notable that when GSNO was added to HEK293-OATP1B1 cells, the GSH content increased faster than in HEK293 cells, which may indicate that GSH is a substrate for OATP1B1. GSH is unable to penetrate through the cell membrane, and specific transporters are required for its transmembrane transport [37]. The ability of GSH to be transported by OATP1B1 revealed in our study may be another type of its transport into hepatocytes. The obtained results also confirm the previously expressed assumption that GSNO is transported through the cell membrane indirectly, by first transferring the nitroso group from GSNO to another thiol-containing amino acid [1].

The study has also shown that the increase in the NO level in HepG2 cells caused by GSNO leads to an increase in the expression of the *SLCO1B1* gene, the level of the OATP1B1 protein, and accelerates the transport of the OATP1B1 substrate atorvastatin into the cells. It is remarkable that the increase in the transport of atorvastatin into HepG2 cells is not observed at all concentrations of GSNO that have caused the increase in protein expression. It can be assumed that at high concentrations, NO causes nitrosylation of OATP1B1, which leads to a decrease in activity. This assumption is confirmed by an increase in the level of nitrosylation products (3-nitrotyrosine and bityrosine). The effects of GSNO may depend on the concentration and time of exposure. When adding GSNO to the immortalized preadipocyte 3 (T3-L1) cell line, it has been shown that concentrations of the NO donor ≥ 500 μM exhibit a toxic effect: cell viability, protein, and triacylglyceride concentrations decrease. Concentrations below 500 μM lead to the initiation of the nitrosylation process, which is an important physiological regulatory mechanism for the maturation of fat cells [38]. A GSNO concentration of 50 μM also exhibits a regulatory effect: when treating splenic B cells, the number of interleukins formed increases [39]. Therefore, GSNO concentrations from 1 to 100 μM have a regulatory effect [39,40], and concentrations above 500 μM are toxic [38]. On the other hand, atorvastatin is a substrate for other transporters, for example, OATP1B3 [41]. Therefore, changes in its uptake may be associated with them.

At the final stage of the study, the mechanisms of the up-regulation of OATP1B1 by GSNO were studied. The most studied action of NO is through cGMP [6]. It was shown that inhibition of the NO-cGMP signaling pathway suppressed the inducing effect of GSNO at all concentrations and all exposure times. The results indicate that the effect of GSNO on OATP1B1 is realized through the NO-cGMP signaling pathway.

The inhibition of Nrf2 also partially neutralized the effect of GSNO on OATP1B1. The study found that GSNO activates Nrf2 (its level in the cell nucleus increases), and inhibition of the NO-cGMP signaling pathway has suppressed the inducing effect of GSNO on Nrf2. The obtained results indicate that GSNO activates the NO-cGMP signaling pathway, which activates Nrf2, which in turn can increase the expression of OATP1B1.

FXR had no effect in the up-regulation of OATP1B1; on the other hand, LXR*a* took part in the action of GSNO at 100 µM with exposure for 72 h. It can be assumed that the released products of nitrosative stress activated LXR*a*.

The obtained results have important practical significance. In clinical practice, NO donors, nitrates, and OATP1B1 substrates, such as statins (cholesterol-lowering drugs), are often prescribed together [42]. Up-regulation of the OATP1B1 by NO may lead to accelerated uptake of statins into hepatocytes, where they exert their pharmacological effect. In the future, it is planned to test the affiliation of GSNO to substrates and modulators of other clinically significant transporters.

## 5. Conclusions

GSNO is not a substrate of OATP1B1 but stimulates its expression and activity. Upregulation of OATP1B1 by GSNO is carried out through the NO-cGMP, Nrf2, and LXR*a*.

## Figures and Tables

**Table 1:** The effect of GSNO on the intracellular transport of OATP1B1 substrate atorvastatin in HepG2 (pmol/mg/min, M ± SD, n = 3).

Rifampicin/GSNO Concentrations	Rifampicin	Duration of Incubation with GSNO		
		3 h	24 h	72 h
Control	4.78 ± 0.49	4.95 ± 0.52	4.85 ± 0.29	4.65 ± 0.41
1 μM	4.52 ± 0.13	5.07 ± 1.67	4.62 ± 0.36	4.79 ± 0.52
10 μM	3.63 ± 0.32 **	4.69 ± 0.69	6.11 ± 0.13 *	6.95 ± 0.99 #
50 μM	3.39 ± 0.35 **	5.12 ± 1.17	6.76 ± 0.77 **	7.20 ± 1.55 *
100 μM	2.93 ± 0.34 ***	3.95 ± 0.39	5.82 ± 0.41 #	5.88 ± 1.28

# *p* < 0.1, * *p* < 0.05; ** *p* < 0.01; *** *p*< 0.001—differences with the control, ANOVA, post-hoc Dunnett’s test. (M ± SD, n = 3). Rifampicin—OATP1B1 inhibitor was used as a positive inhibition control with 15 min pre-incubation.

## Data Availability

The data presented in this study are available on reasonable request from the corresponding author.

## Supplementary Materials

The following supporting information can be downloaded at: https://www.mdpi.com/article/10.3390/biom15030428/s1, File S1: Original Western Blotting Figures.
