# Supplementary material for: S-Nitrosoglutathione Is Not a Substrate of OATP1B1, but Stimulates Its Expression and Activity
Source: Biomolecules. 2025 Mar 17;15(3):428. doi: 10.3390/biom15030428 (PMC11940419; doi:10.3390/biom15030428)

Figure 4 (**a**)

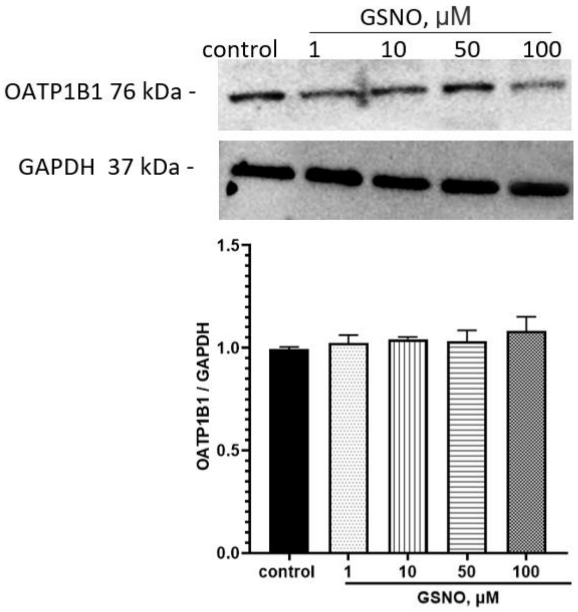

Figure 4 (**b**)

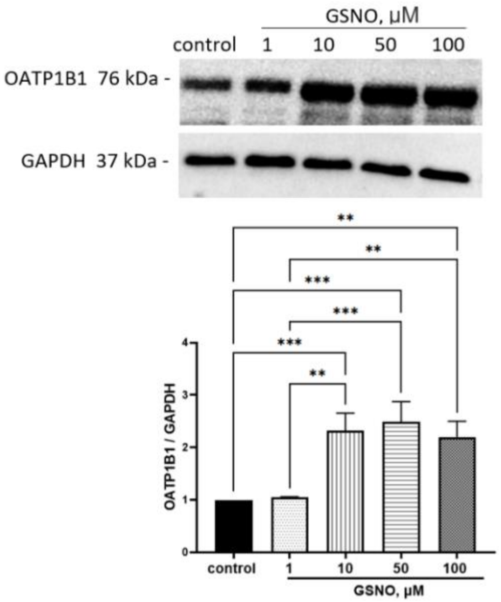

Figure 4 (**C**)

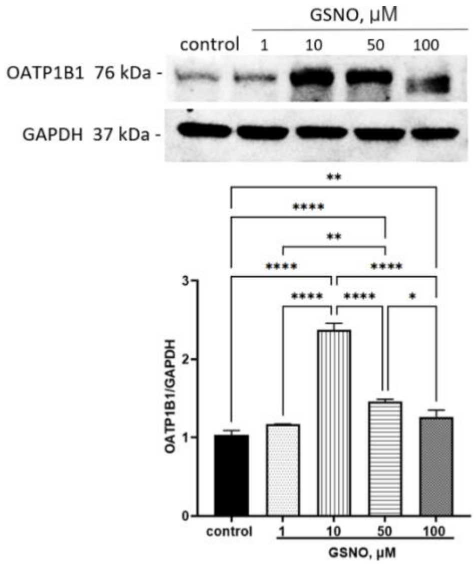

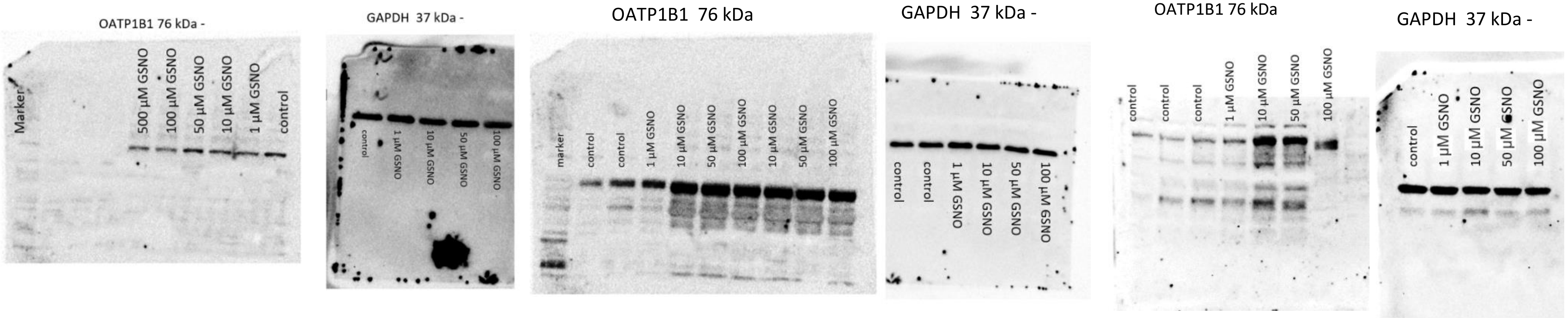

Figure 5 (a)

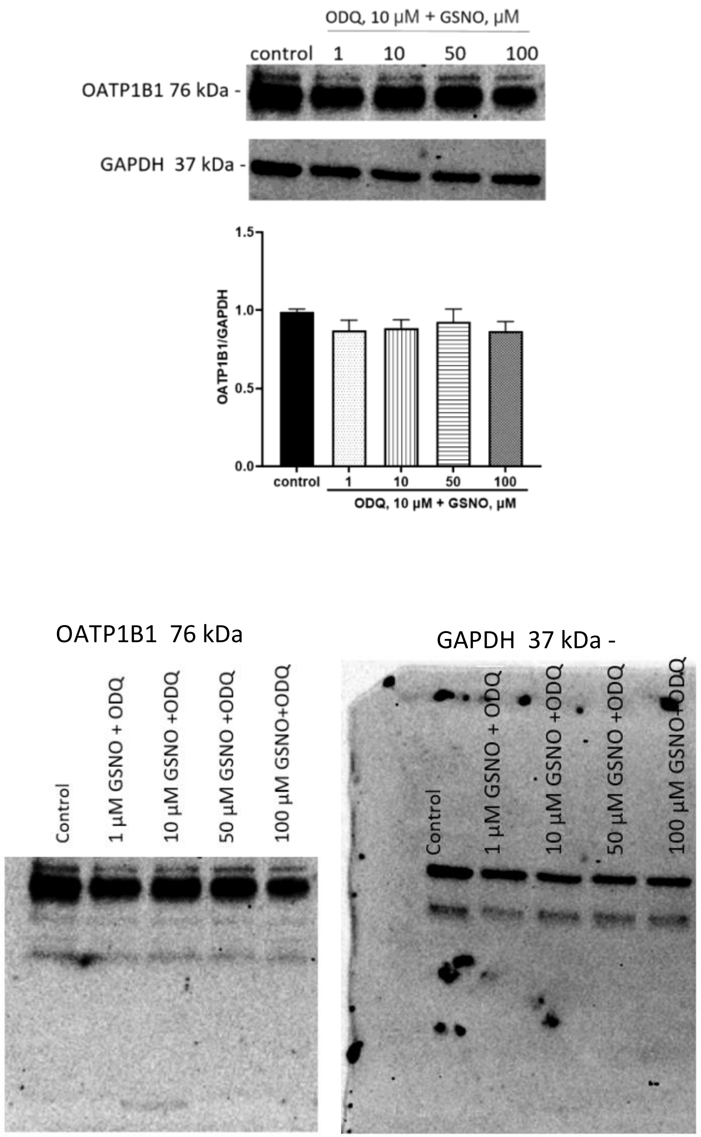

Figure 5 (b)

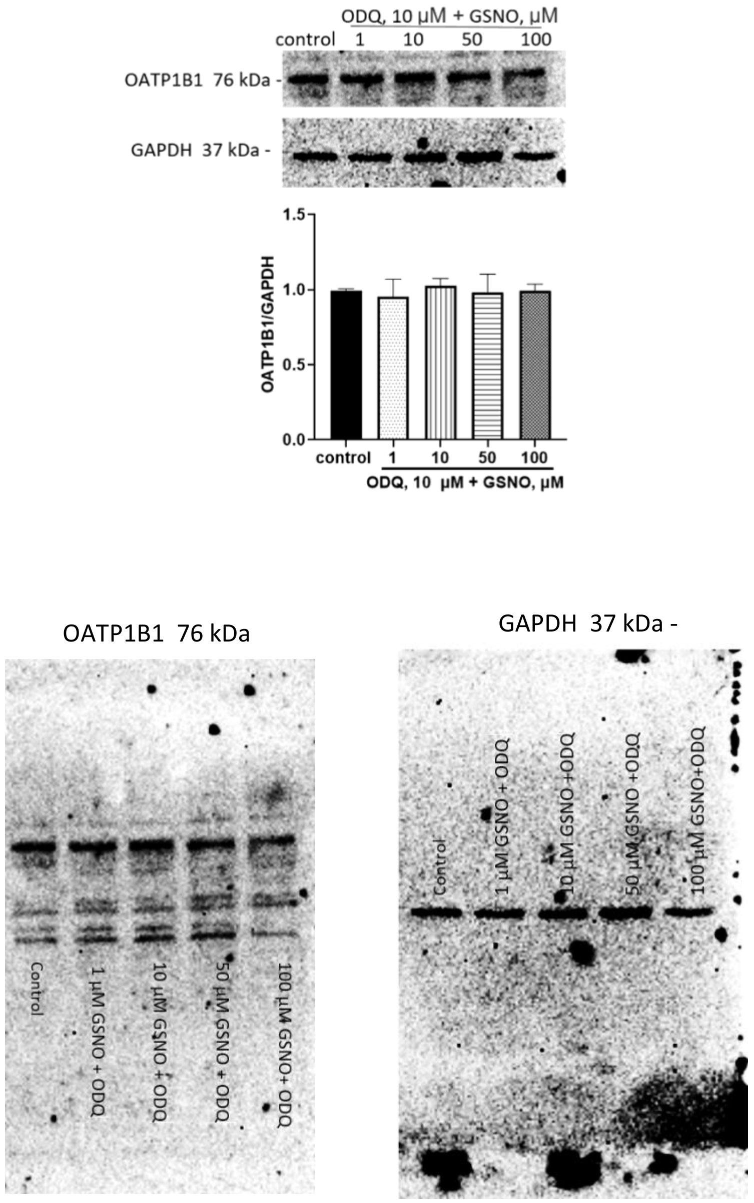

Figure 6(a)

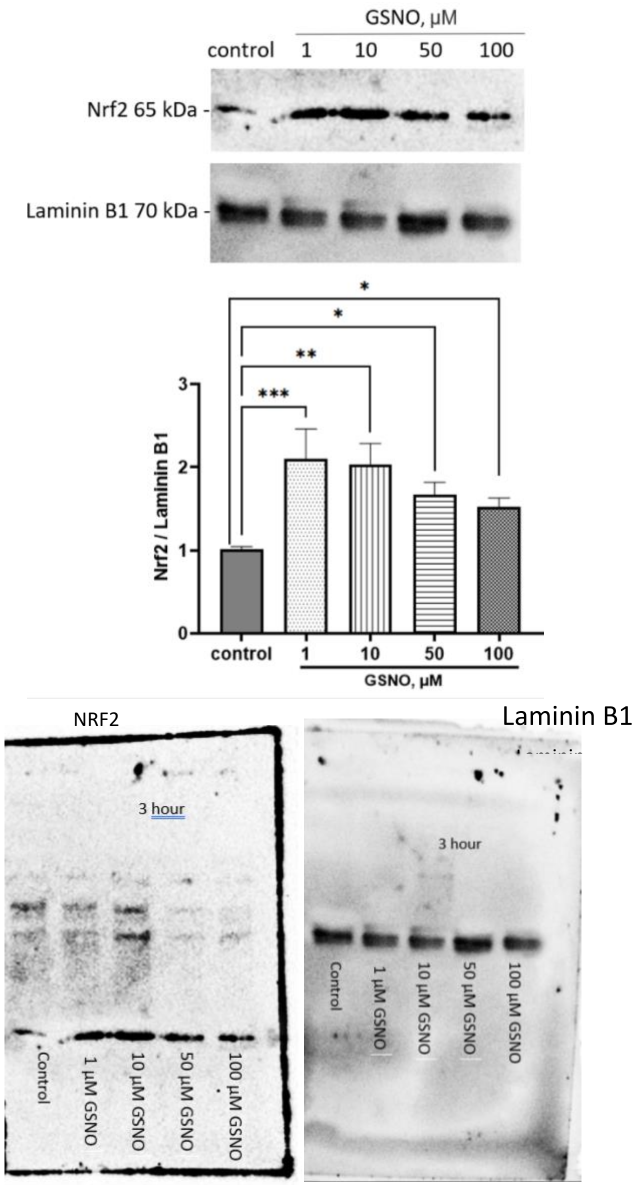

Figure 6 (**b**)

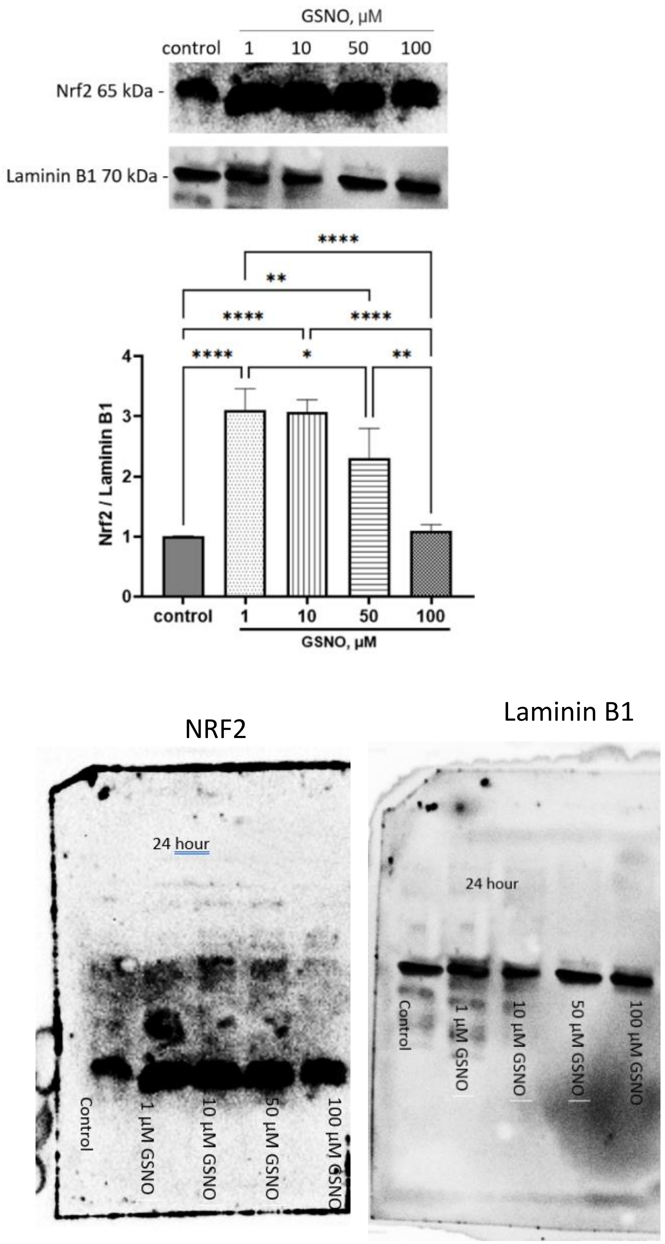

Figure 6 (**C**)

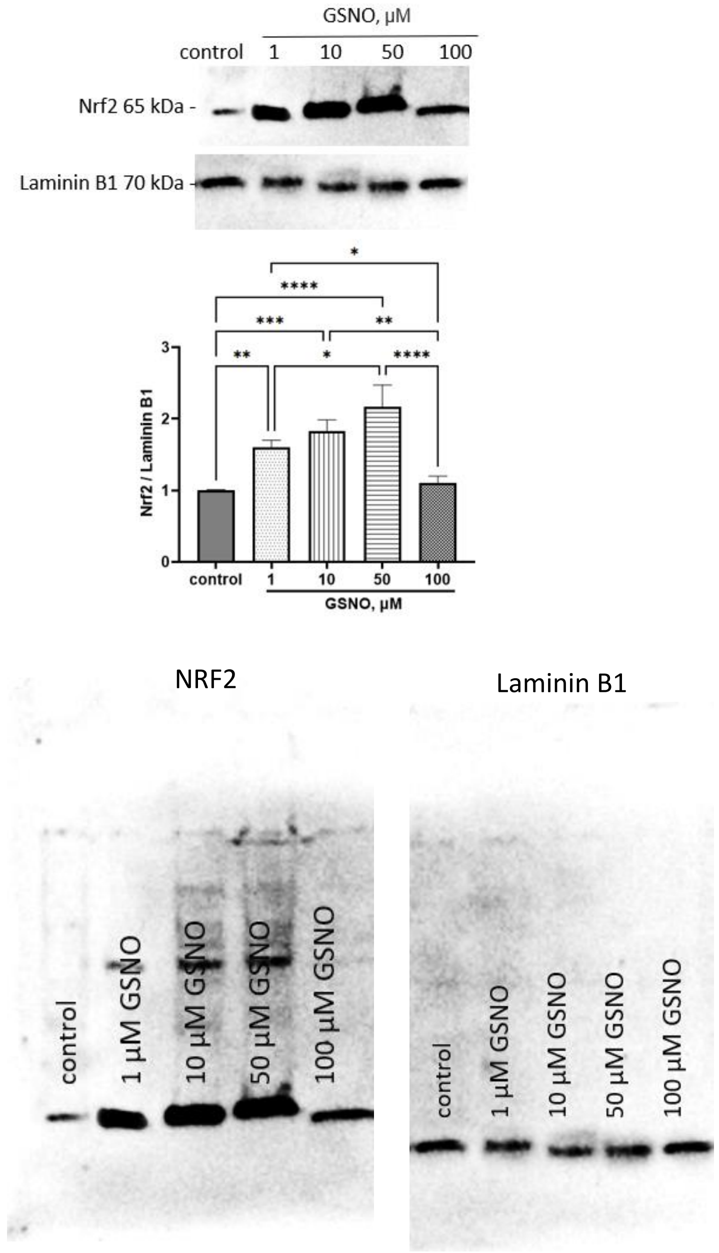

Figure 7(a)

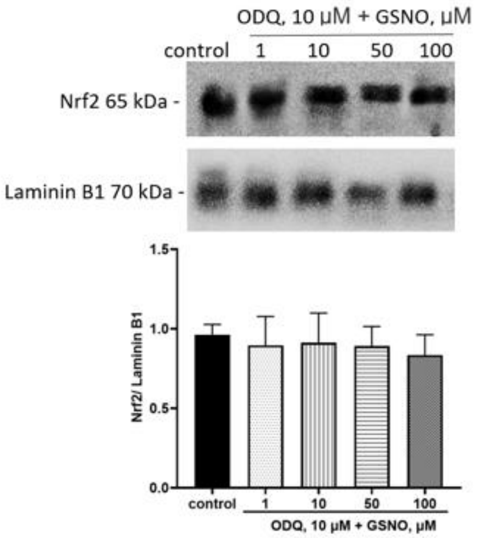

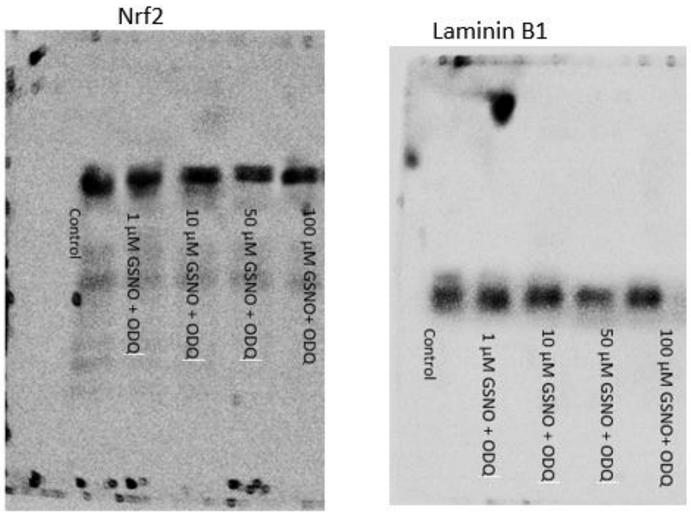

Figure 7 (**b**)

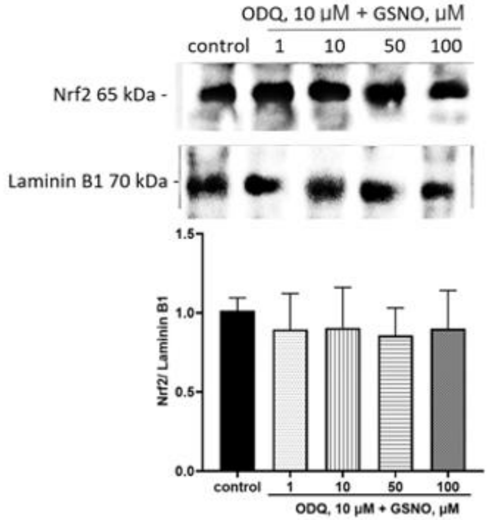

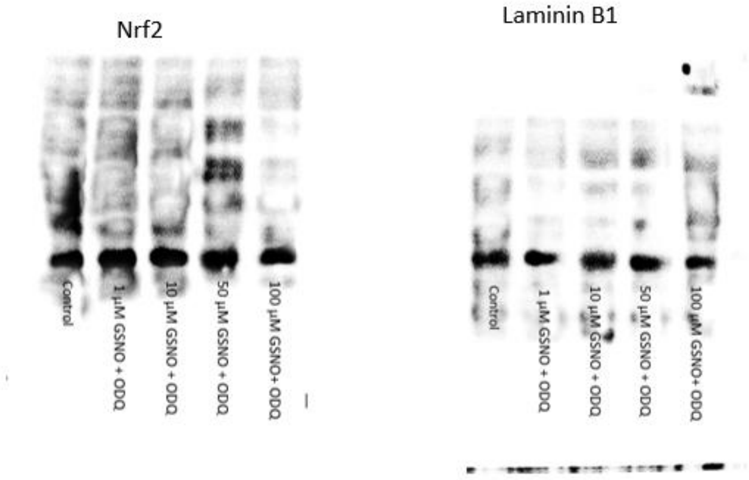

Figure 8(a)

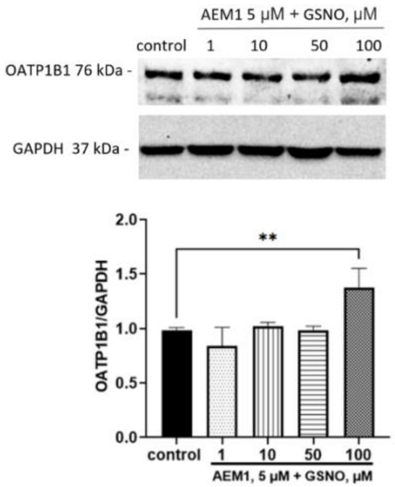

Figure 8 (**b**)

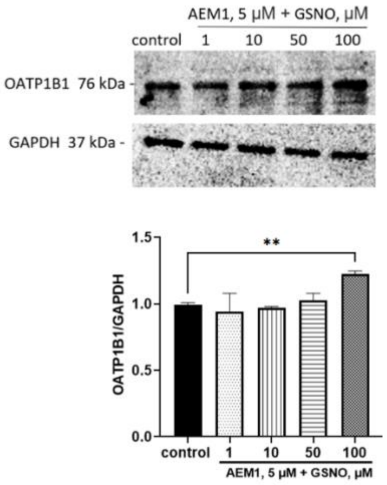

OATP1B1 76 kDa

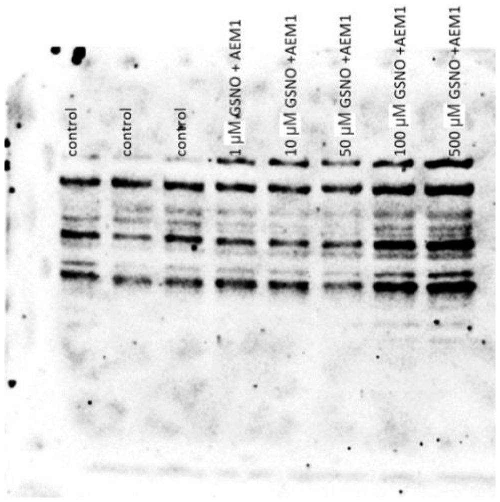

GAPDH 37 kDa -

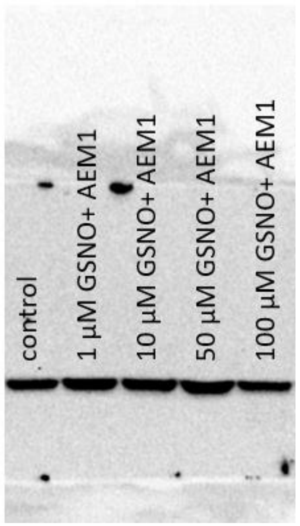

OATP1B1 76 kDa

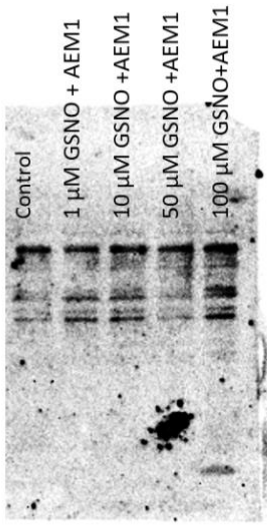

GAPDH 37 kDa -

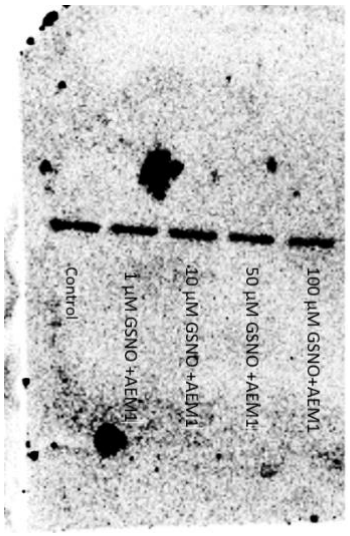

Figure 9 (a)

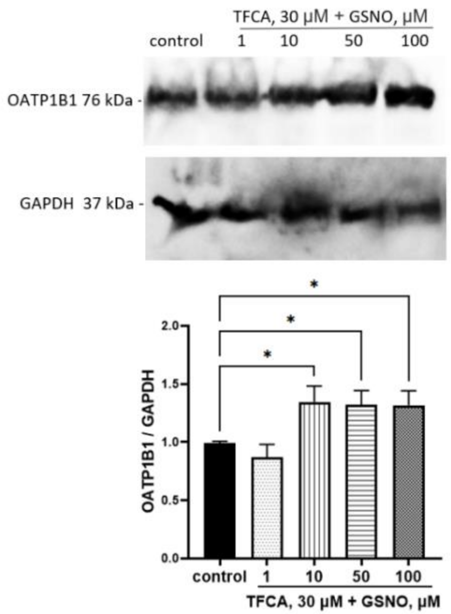

Figure 9 (b)

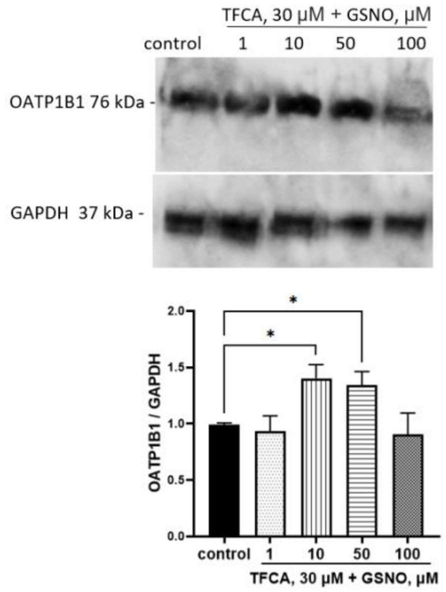

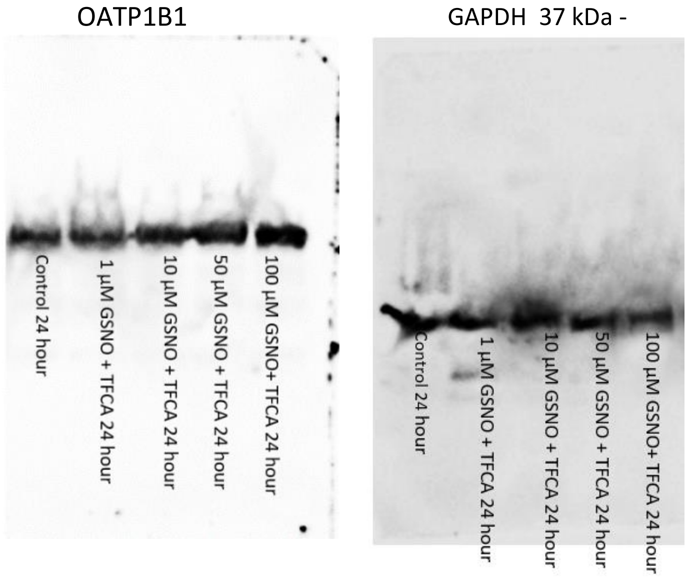

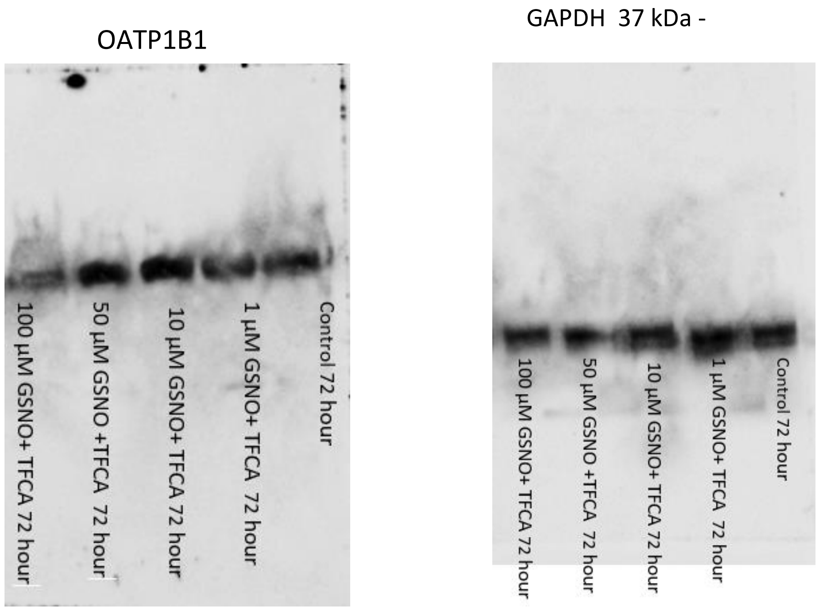

Figure 10 (**a**)

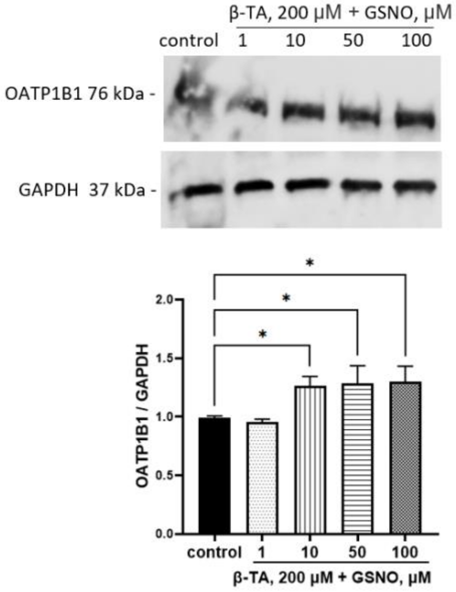

Figure 10 (**b**)

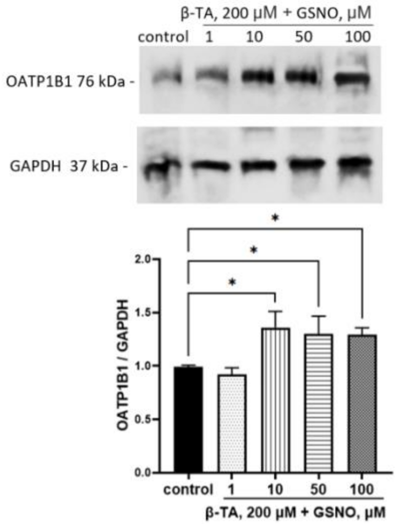

OATP1B1

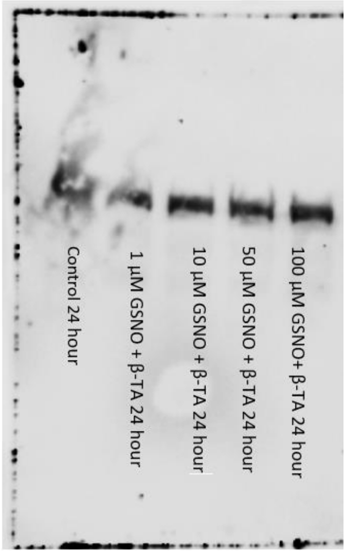

GAPDH 37 kDa -

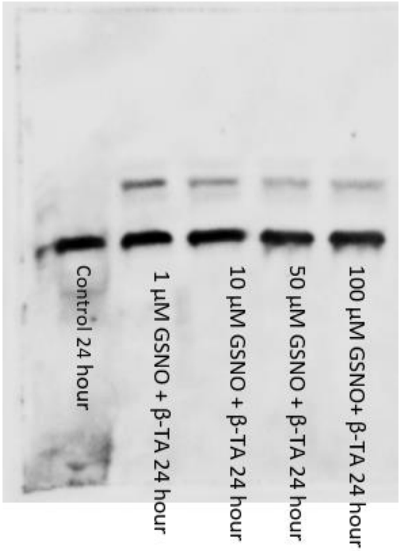

OATP1B1

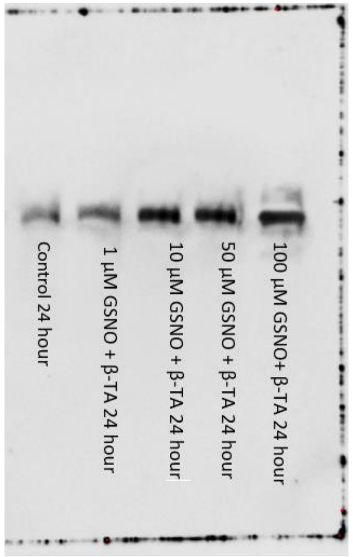

GAPDH 37 kDa -

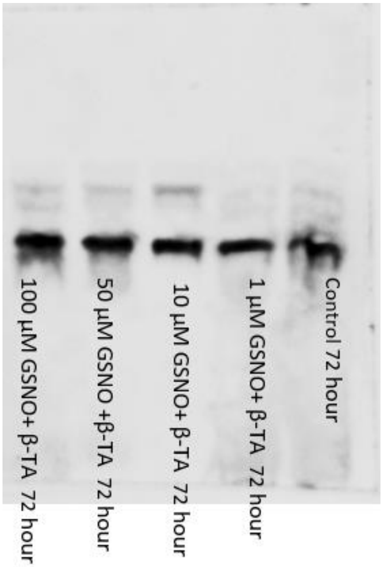

Figure 11 (a)

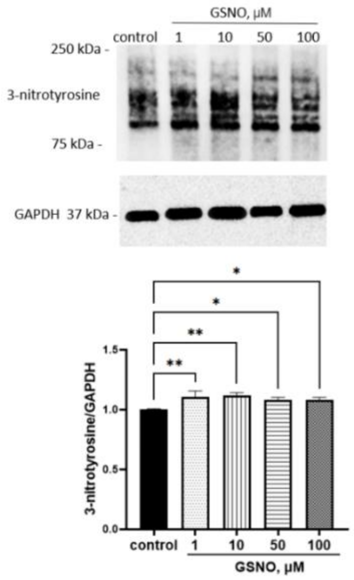

Figure 11 (b)

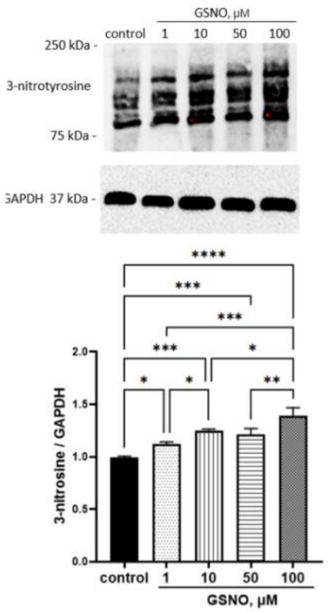

Figure 11 (c)

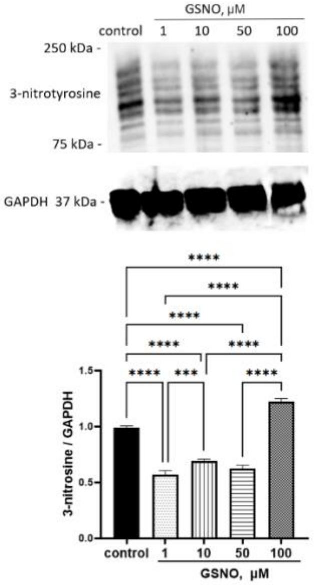

3-nitrotyrosine

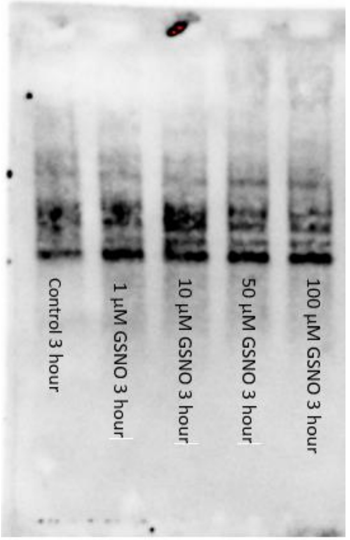

GAPDH

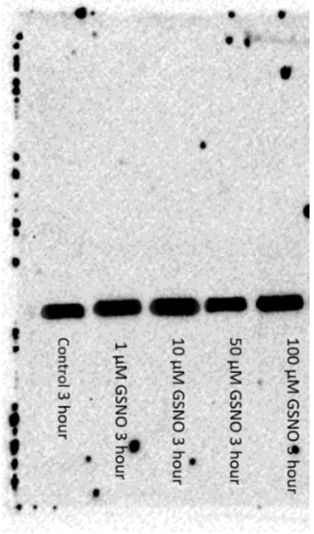

3-nitrotyrosine

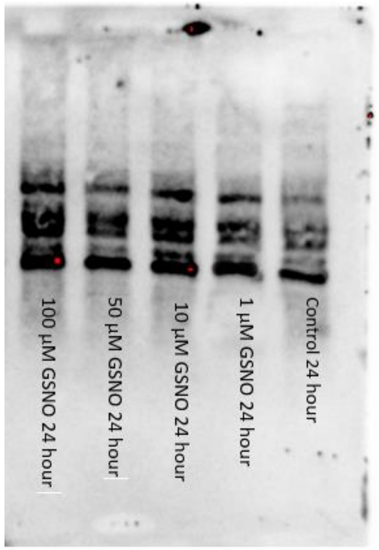

GAPDH

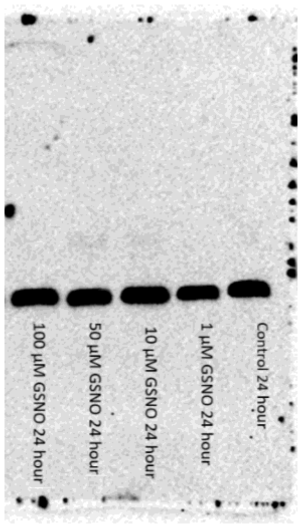

3-nitrotyrosine

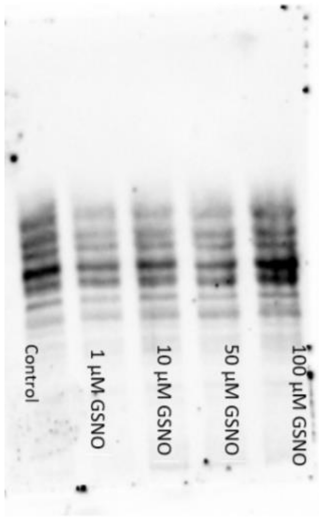

GAPDH

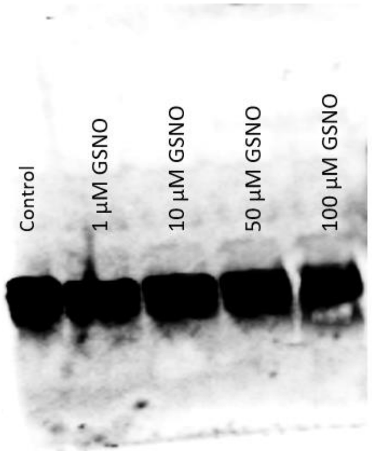

Supplement: Supplementary file 1 [file biomolecules-15-00428-s001.zip › File S1. Original Western Blotting Figures.pdf]
